# Gambling and its associated factors among students of the University of Ilorin, Nigeria

**DOI:** 10.4102/sajpsychiatry.v32i0.2481

**Published:** 2026-01-28

**Authors:** Oludolapo Oladeji, Mosunmola F. Tunde-Ayinmode, Amudalat T. Kuranga, Adebusola J. Ogunmodede, Dauda Sulyman

**Affiliations:** 1 Department of Behavioural Sciences, University of Ilorin Teaching Hospital, Ilorin, Nigeria; 2 Department of Behavioural Sciences, Federal Neuropsychiatric Hospital, Budo-Egba, Kwara State, Nigeria; 3 Department of Behavioural Sciences, Faculty of Clinical Sciences, University of Ilorin, Ilorin, Nigeria

**Keywords:** gambling, sport betting, gambling disorder, university students, Nigeria

## Abstract

**Background:**

Studies have revealed a global surge in gambling and gambling disorders, particularly among young adults, including university students. This disturbing trend poses significant risks not only to individual’s mental well-being but also to the wider community.

**Aim:**

The objectives of the study were to determine the prevalence of gambling, gambling disorder and the associated socio-demographic factors among undergraduate students of University of Ilorin.

**Setting:**

The study was conducted at the University of Ilorin, a federal government owned tertiary educational institution located in Kwara State, North-Central Nigeria.

**Methods:**

A pro forma questionnaire was used to obtain socio-demographic information about the students. Respondents who had gambled in their lifetime proceeded to complete the self-administered South Oaks Gambling Screen (SOGS). Score of at least 1 out of 20 on the SOGS indicated gambling disorder, having met the criteria for either problem gambling (score 1-4) or pathological gambling (score ≥ 5).

**Results:**

Responses of 2044 of the respondents were analysed. Their mean age was 21.74 ± 2.64 years, and male respondents accounted for 54.5%. A total of 409 (20%) of respondents had ever gambled in their lifetime, with sports betting been the most gambled activity. About 14.9% of the respondents had gambling disorders (10.5% problem gambling; 4.4% pathological gambling). Male gender, positive family history of mental illness, a lack of adequate financial support and staying off-campus were significantly associated with presence of gambling disorders. Logistic regression revealed that only positive family history of mental illness was predictive of gambling disorder (OR = 6.987, 95% CI [2.119–23.038], *p* = 0.001).

**Conclusion:**

This study revealed that three-quarters of respondents with a lifetime history of gambling developed gambling disorder.

**Contribution:**

This implies that a significant proportion of individuals who initially engage in social gambling may have a propensity to develop gambling disorder overtime.

## Introduction

The term gambling refers to the act of playing a game for money or property, making a bet on an uncertain outcome, or taking something on a contingency.^1^ It involves placing something of value at-risk with hopes of gaining something of greater value.^2^ Activities that involve gambling include instant lotteries, bingo, betting on billiards or pool, card games, sports betting, casino games, video lottery terminals, internet gambling and dice throwing.^3^ The National Sport Lottery Commission, the body saddled with the responsibility of regulating gambling or lottery in Nigeria defines sport betting as activity involving the prediction of sporting results and placing a bet on its outcome in anticipation of winning a prize.^4^ Over the years, many gambling companies have sprung up in Nigeria, which include Betnaija, Naijabet, 1XBet and many others.

Gambling is a global phenomenon with different level of acceptance in different societies. Some culture and religion frown at the act of gambling, which might explain the lower prevalence in those environments, while some cultures are more tolerant.^5^ In Australia, Dowling et al.^6^ found a national prevalence of gambling participation to be 63.9%. Another Australian study estimated gambling participation to be between 61.2% and 72.8% using the Problem Gambling Severity Index (PGSI) among the general population in Adelaide, Australia.^7^ In a study among KwaZulu-Natal people of South Africa, a prevalence rate of lifetime gambling was found to be 68%, while 11% of those who gamble met the criteria for gambling disorder.^8^ In a study in Uganda, East Africa, about 75.7% of Ugandans had participated in gambling-related activities in 12 months preceding the survey.^9^ In a survey involving close to 4000 youths from Kenya, Tanzania, Nigeria, Ghana, South Africa and Uganda, about 54% of the youths were involved in gambling-related activities such as football betting, betting on animals, and casino games. Of these nations, Kenya recorded the highest prevalence with 76% involved in gambling, while Ghana had the least with 42%.^10^ In a study in Southwestern Nigeria by Bankole,^11^ 73.64% of the sampled population of 320, majority of whom were youths were involved in gambling.

The Diagnostic and Statistical Manual of Mental Disorders, Fifth Edition (DMS-5) adopted the term gambling disorder and classified gambling disorder under substance-related and addictive disorders because it has clinical features that are similar to substance use disorder.^12^ Gambling disorder or disordered gambling as used by DSM-5 is an ‘umbrella’ term that accommodates every gambling-related problem including ‘pathological gambling’, which is graded as a severe form of disordered gambling and problem gambling, which is less severe in the spectrum.^13,14^ In Australia, Dowling et al.^6^ reported a prevalence of 0.4% for problem gambling and 4.9% for at risk gambling in the general population. The diagnosis of problem gambling is made when 3–4 symptoms of the 10 criteria are present in an individual.^13,14,15^ However, the Victorian Casino and Gaming Authority (VCGA), Australia argued that the presence of harms rather than symptom counts should be used to define problem gambling. Calado and Griffiths^16^ found in their global survey that the prevalence of problem gambling ‘in the past year’ in North America ranged from 2% to 5%, in Europe 0.1% to 3.4%, in Asia 0.5% to 5.8% and Oceania 0.4% – 0.7%. They attributed differences in the prevalence rates as because of variations in the use of gambling screening instruments and their scoring systems. Potenza et al.,^17^ also observed that some of the screening tools could give a false high prevalence.

A very important factor that might contribute to high rates of gambling is the versatility of the youths with Information and Communication Technology.^18^ South Africa, Kenya and Nigeria have approximately 11%, 8% and 7% of their populations, majority of whom are youths, actively using the internet.^19^ Accessibility to betting platforms and gambling websites on mobile phones might then be very easy, and individual identity would be protected from the public eye.

Levels of involvement in gambling have been described to include the social gambling, which is considered to be a benign form of entertainment, often occurring during festive periods such as weddings and birthdays. It mainly happens amid friends, colleagues and relatives, and only lasts for a few hours and not driven by the gains expected from gambling.^20^ Social gambling is devoid of ‘loss of control’, which differentiates it from pathological gambling.^21,22^ The next level of involvement is low-risk gambling, which describes people who reported some episodes of gambling in the past year but did not meet any of the DSM-V diagnostic criteria for pathological gambling. At-risk gambling or ‘in-transition’ consists of individuals involved in gambling participation who may later develop Problem or Pathological gambling.^19,23,24^

Factors such as early exposure to gambling, easy accessibility to gambling venues and increased gambling options have been identified to be associated with the development of gambling disorder. Early exposure to gambling has been found to increase the likelihood of developing gambling problems later in life.^25^ Furthermore, easy access to gambling opportunities was associated with higher rates of gambling participation and problem gambling.^26^ The increased availability of gambling options, both online and offline, contributes to the ease with which individuals can engage in gambling activities. Peer influence has also been identified as a risk factor for gambling behaviours among youths as they are more likely to engage in gambling activities if they observe their peers doing so.^27^ Peer pressure and the desire for social acceptance contribute to the initiation and continuation of gambling behaviours among young individuals. Also, certain personality traits such as impulsivity, sensation-seeking and a lack of self-control have been linked to an increased susceptibility to gambling problems.^28^ Individuals with these traits may be more prone to seeking the excitement and thrill associated with gambling, making them susceptible to developing gambling disorders. Cultural and social factors are other risk factors that play roles in shaping attitudes towards gambling and influencing the prevalence of gambling-related problems. In societies where gambling is widely accepted and culturally ingrained, individuals may be more likely to engage in gambling activities.

It is observed that there is dearth of studies on gambling and gambling disorders in this part of the country especially among undergraduate students. Therefore, this study aimed to identify gambling disorder and its associated factors among students of University of Ilorin, Nigeria, with specific objectives, which were to determine the prevalence of and socio-demographic factors associated with gambling disorders among students of University of Ilorin.

## Research methods and design

### Study’s setting and location

The study was carried out at the University of Ilorin, which is a Federal Government-owned tertiary institution of education in Kwara State, North-Central geopolitical zone, Nigeria. University of Ilorin has 15 faculties with 94 academic departments. The total undergraduate enrolment for the 2018/2019 academic year was 44 919 students. The duration of undergraduate degree programmes ranges from 3 years to 6 years depending on the mode of entry and the course of study.

### Study design

This study was a school-based cross-sectional study.

### Study population

All registered and consenting undergraduate students of the University of Ilorin for the 2019/2020 academic session constituted the study population. The sample size was 2150 undergraduates cutting across all faculties.

### Sampling method

The subjects of this study were selected from the 15 faculties of the University using a multistage sampling technique. A simple random sampling method using ballot was used to select 25% of the departments in each of the 15 faculties. Proportional allocation technique was used to determine the number of students who were recruited from each department. Within each department, one compulsory course was randomly selected from the list of compulsory courses for each academic level, where respondents were addressed regarding the conduct of the study. Subjects were stratified based on gender to allow for proportional representation of male and female students. This was performed using the male-female representation per faculty. This sampling method has been used by Sajo^29^ in his study on substance abuse among undergraduate students of the University of Ilorin.

### Inclusion and exclusion criteria

The main inclusion criterion was being an undergraduate student of the University of Ilorin. Those excluded from study were those who refused to give their consent and those who are not mentally or physically fit to participate in the study.

### Instruments

Data collection was carried out by the following instruments.

#### Pro forma questionnaire

This was designed by the researchers to obtain the socio-demographic variables such as age, gender, faculty, department, academic level, marital status and religion.

#### South Oaks Gambling Screen

The South Oaks Gambling Screen (SOGS) is a validated and reliable instrument for detecting gambling problems. Developed in 1987 by Lesieur and Blume,^30^ the SOGS was found to be highly correlated with scores on the instrument that detect pathological gamblers.^31,32^ The revised version that was used by the researchers comprises 16 items with 4 sub-questions, making it twenty questions. A score of 0 means no problem, 1–4 indicates some problems, while a score of 5 or more indicates probable pathologic gambling. Reliability was confirmed through Cronbach’s alpha and test-retest correlation, and a value of 0.97 and 0.71 were obtained, respectively.^30^

### Procedure for the main study

The number of departments sampled was 25% of the number of departments per faculty. Questionnaires were administered to the recruited students during a compulsory lecture after the nature and purpose of the research had been duly explained to them. Exclusion criteria were expressly stated before the questionnaires were administered. Students who did not consent to the study, as well as those who were excluded were separated from the participants to prevent communication and undue distractions from these categories of students.

Data collection commenced in January 2021 and ended in May 2021. The pro forma and the SOGS were self-administered. Any respondent without gambling history did not proceed to fill the SOGS questionnaire.

### Data analysis

Completed questionnaires were sorted out and coded serially. Raw data were first imputed into the Statistical Package for Social Sciences version 25, which was used for data analyses. Distribution of socio-demographic variables was analysed with the use of percentages. Associations between gambling disorder and socio-demographic variables were compared using Chi-square and where necessary logistic regression was applied to determine the predictors of gambling disorder. A statistically significance level of less than 0.05 was used.

### Ethical considerations

Ethical clearance to undertake the study was obtained from the Ethics and Research Committee of the University of Ilorin Teaching Hospital (UITHERC) with approval number NHREC/02/05/2010. Approval was sought and obtained from the Dean of Student Affairs of the University of Ilorin for the conduct of the study. Permission was granted from departmental heads and lecturers to use their classes for the administration of the questionnaire. The students were duly informed about the study, and those who gave their written informed consent were recruited for the study.

## Results

### Socio-demographic characteristics of respondents

A total of 2044 out of 2150 questionnaire were analysed, giving a response rate of 95.1% for the study. The mean age of the respondents was 21.74 (± 2.64) years. Majority of them were in the age group 20–24 years, which accounted for 64.1% followed by those aged between 15 years and 19 years, which were 434 (21.2%). Respondents who were single accounted for 98.5%. Male respondents accounted for 54.5%. Of the 15 faculties of the institution, the faculty of Education accounted for one-fifth of the population of respondents while veterinary medicine, was the least in terms of representation and accounted for about 0.3% of the study population. The 200 level classes had slight preponderance of 557 (27.1%), while 500 level classes accounted for the least 66 (3.2%). A total of 1859 students (90.5%) were satisfied with their academic performance, while 41 (2.0%) were undecided. A total of 858 (42%) students lived within the hostels, 146 (16.9%) lived with their parents, while the remaining lived in rented apartments. Of those who lived in the hostels, 57.7% lived on-campus, while 42.3% lived off-campus hostels. About 75% were supported financially by their parents, while as few as 55 (2.7%) had other sources of financial support other than self-support and support from other family members. Majority of the respondents (65.1%) were from monogamous family setting. A total of 1413 (69.1%) were satisfied with their social support (Table 1).

**TABLE 1:** Socio-demographic variables of the respondents.

Variables	*n*	%
**Age (years)**		
15–19	434	21.2
20–24	1311	64.1
≥ 25	299	14.7
**Gender**		
Female	929	45.5
Male	1115	54.5
**Religion**		
Christianity	922	45.2
Islam	1117	54.6
Others	5	0.2
**Marital status**		
Single	2014	98.5
Married	30	1.5
**Family history of mental illness**		
No	11	-
Yes	2033	-
**Academic satisfaction**		
Satisfied	1859	90.5
Not Satisfied	154	7.5
Undecided	41	2.0
**Accommodation type**		
Hostel	858	42.0
Rented apartment	840	41.1
Live with parents	346	16.9
**Location of hostel (*N* = 858)**		
On-campus	495	57.7
Off-campus	363	42.3
**Sources of financial support**		
Self-support	152	7.4
Parents	1533	75.0
Other family members	304	14.9
Other sources	55	2.7

### Prevalence of gambling among respondents and responses on the South Oaks Gambling Screen

Of the 2044 respondents, 409 (20%) had a lifetime history of gambling, who responded ‘yes’ to the question ‘have you ever gambled’. Responses on the SOGS revealed that out of the 409 respondents who had ever gambled, 105 had no gambling problem, although they scored zero on the SOGS. Of the 409 respondents, 214 (10.5%) had ‘problem gambling’ score between 1 and 4 on the SOGS screen, while 90 (4.4%) had ‘pathological gambling’ score of minimum 5 on the SOGS, giving a prevalence rate of 14.9% as those having gambling disorder (Table 2).

**TABLE 2:** South Oaks Gambling Screen scores of the respondents involved in gambling (*N* = 409).

SOGS scores	Frequency	%
No problem gambling (score 0)	105	25.7
Problem gambling (score 1–4)	214	52.3
Pathological gambling (score ≥ 5)	90	22.0

SOGS, South Oaks Gambling Screen.

### Relationship between demographic characteristics of respondents and gambling disorder

A comparison of age groups showed there were more cases of gambling disorder in respondents of age 25 years and beyond, which represents 54 (18.1%) compared to other age groups, however, it was not statistically significant (*χ*^2^ = 5.837, *p* = 0.054). There were statistically significant positive associations between gambling disorder and male gender, those with positive family history of mental illness, those living at off-campus hostel, and the respondents who had to support themselves financially. There was no statistical significance between the marital status, religion, family setting and level of academic satisfaction (Table 3).

**TABLE 3:** Relationship between socio-demographic characteristics and gambling disorder.

Variables	Gambling		Disorder		*χ* ^2^	*p*-value

	Non-cases		Cases			

	*n*	%	*n*	%		
**Age (years)**						
15–19	51	11.8	383	88.2	5.84	0.054
20–24	199	15.2	1112	84.8	-	-
≥ 25	54	18.1	245	81.9	-	-
**Gender**						
Female	116	12.5	813	87.5	7.66	0.006*
Male	188	16.9	927	83.1	-	-
**Religion**						
Christianity	135	14.6	787	85.4	0.13Y	0.937
Islam	168	15.0	949	85.0	-	-
Others	1	20.0	4	80.0	-	-
**Marital status**						
Single	302	15.0	1712	85.0	1.03Y	0.311
Married	2	6.7	28	93.3	-	-
**Family history of mental illness**						
Yes	6	54.5	5	45.5	13.75	0.001*
No	298	14.7	1735	85.3	-	-
**Academic satisfaction**						
Satisfied	275	14.9	1574	85.1	0.00	0.999
Not satisfied	23	14.9	131	85.1	-	-
Undecided	6	14.6	35	85.4	-	-
**Accommodation type**						
Hostel	128	14.9	730	85.1	0.06	0.970
Rented apartment	126	15.0	714	85.0	-	-
Live with parents	50	14.5	296	85.5	-	-
**Location of hostel (*N* = 858)**						
On-campus	65	12.6	452	87.4	5.64	0.018*
Off-campus	63	18.5	278	81.5	-	-
**Sources of financial support**						
Self-support	37	24.3	115	75.7	15.42	0.001*
Parents	226	14.7	1307	85.3	-	-
Other family members	32	10.5	272	89.5	-	-
Other sources	9	16.4	46	83.6	-	-

* , Statistically significant at *p* < 0.05.

### Predictors of gambling disorders

All variables with statistically significant association at the univariate analysis were subjected to logistic regression analysis to ascertain possible predictors of gambling disorder (Table 4). An outcome of each variable was used as a reference category against which other outcomes of the same variable were compared. Male gender and staying at the off-campus hostel were significant predictors of gambling disorder with *p*-values 0.006 and 0.018, respectively.

**TABLE 4:** Predictors of gambling disorder among respondents.

Variable	B	OR	CI		*p*-value
			Lower	Upper	
**Gender**					
Female	−0.35	0.70	0.55	0.90	0.006*
Male REF	-	-	-	-	-
**Family history of mental illness**					
No REF	-	-	-	-	-
Yes	1.94	6.99	2.12	23.04	0.001*
**Financial support**					
Self-support REF	-	-	-	-	-
Parents	0.48	1.61	0.63	4.15	0.320
Other family members	−0.21	0.81	0.35	1.88	0.626
Other sources	0.71	0.49	0.19	1.25	0.137
**Location of hostel**					
Off-campus REF	-	-	-	-	-
On-campus	−0.45	0.63	0.43	0.92	0.018*

B, coefficient of logistic regression; OR, odds ratio; CI, confidence interval; REF, Reference category.

* , Statistically significant at *p* < 0.05.

## Discussion

### Socio-demographic characteristics of respondents

This study showed more male respondents 55.5% than female 44.5% which is reflective of the enrolment profile of 27 914 (62.1%) male and 17 005 (37.9%) female students for the year 2018/2019 academic session. Over the years, although female enrolment in the university has increased, there is still a relatively predominant number of male students.^33^ Majority of students lived in hostels and rented apartments around the university, which accounted for 42.0% and 41.1%, respectively. Although the University of Ilorin authority provided some accommodation within the campus, these were grossly inadequate, hence the need for some students to opt for rented apartments close to the campus. Students generally prefer to be close to the academic environment as this is believed to help them in their academic pursuits and ease any potential difficulties that might result from transportation to and from the campus.

### Prevalence of gambling

The lifetime prevalence of gambling in this study was 20%. This is similar to a study in India among college students where a lifetime prevalence of 19.5% was reported.^34^ A recent Nigerian study found a prevalence of 40.3% in a multiregional setting albeit with a self-designed questionnaire.^35^ This high prevalence might not be that reliable because of the fact that instrument used was not standardised. The findings in our study, likewise, differ from that found by Oster and Knapp^36^ who surveyed college students at the University of Las Vegas and found the lifetime of gambling to be 97% for male and 91% for female students. The comparably higher prevalence as reported could be because of the differences in sociocultural setting of the study location. Las Vegas is a well-known city for its numerous casinos and gambling industry that offer easy access to gambling opportunities.

### Prevalence of gambling disorder

The prevalence of gambling disorder is 14.9% of the respondents’ population. This is similar to a study by Oyebisi et al.,^37^ which reported 14.2% in southwestern Nigeria. Aderinto et al.^38^ found a prevalence of 10.42% among medical and dental students in Nigeria. The academic rigours of medical and dental schools might have accounted for the slight drop in their study. More staggering was the findings by Afe et al.^39^ who reported a prevalence of 30.5% among a youthful but non-academic population in southwest Nigeria. However, in a meta-analytical review of disordered gambling in the US and Canada, a study found a prevalence of 11.5% for adolescents and 9.5% for college students.^16^

The prevalence of problem gambling which is of a milder severity of gambling disorder was 10.5%. This finding was similar to that found in KwaZulu-Natal region of South Africa where 11% of the general population had problem gambling.^8^ However, it is slightly higher than 7.7% that was reported by Oyebisi et al.^27^ in Nigeria and 7.4% that was reported by Sanju George et al,^28^ in India. A much higher prevalence was reported in a study in Limpopo, South Africa where there is a mix of rural and urban populations, which reported that 23.8% of the 900 respondents in the study were problem gamblers.^40^

Prevalence of pathological gambling, which is a more severe form along the spectrum of gambling disorder was 4.4%. This is close to a prevalence of 5.1% as reported by Yokomitsu et al.^41^ among university students in Japan using the same instrument, SOGS. Furthermore, 4.7% was reported by Gupta and Derevensky^21^ in adolescents in Montreal, Canada. Life-time prevalence of 4.49% was reported by Shaffer et al. in the US and Canada among college students in one of the studies they reviewed.^23^

### Demographic factors associated with gambling disorder

The highest percentage of gambling disorder in this study was found among respondents with age range 25–34 years. This later age bracket could be explained in the light of natural progression of gambling, which is from the period of introduction to the period of gambling and thereafter the onset of the disorder. A Nigerian study reported highest percentage of 78.6% among respondents above 30 years of age.^38^ However, it was not reported if the age range remains dominant at progression to gambling disorder.

In our study, a significant gender difference was observed regarding gambling disorder between both genders. Male participants had more cases of gambling disorder than female participants with 1.5 times more male than female participants. The reason might be that in the African society males tend to engage in more adventurous and risky behaviours that create more financial pressure on them. Even after logistic regression, gender was still significant. This is similar to findings reported by Gupta and Derevensky^21^ that twice as many male participants as female participants gambled on a regular basis and consequently developed gambling disorder. In China, 77.4% male participants compared to 22.6% female participants were found to have gambling disorder among students of Macau University in China.^42^ In the study by Dellis et al.^8^ in South Africa, there was a slight male preponderance in the act of gambling, however, there was no gender difference in the development of gambling disorder.

Living on-campus appeared to be ‘protective’ against gambling disorder than off-campus living. Factor responsible for this could be restrictions of gambling by school authority through prohibition of gambling houses within the school campus. Students who lived off-campus were likely to enjoy more freedom, external influences and as such more prone to gambling, which may later progress to gambling disorder. Another factor found to be associated with gambling disorder was financial support for students during the course of their study. There were more cases of gambling disorder in respondents that financially self-supported themselves compared to those supported by parents or by other family members. Their financial autonomy could make them less accountable to anyone concerning how they spend their money, hence they might not exercise restraints on venturing into gambling. Parental financial support appears to be somewhat protective against gambling disorder, something similarly reported in a United States study by Welte et al.^43^ who reported that more problems with gambling existed amid families with poor socio-economic status in comparison to household with better earnings. It might also mean that students who were well supported financially by their parents had no reasons for finding alternative means of raising money. This study did not find any association between gambling disorder and family setting. It is of note that Odame et al.,^44^ in Ghana found parental divorce and polygamy to be significantly associated with gambling disorder. They attributed this to poverty and reduced parental oversight as often observed in polygamous and divorced homes.

## Conclusion

The study revealed an overall lifetime prevalence of gambling as 20% while that of gambling disorder was 14.9%, comprising of problem gambling (10.5%) and pathological gambling (4.4%). Gambling disorder was associated with male gender, off-campus hostel accommodation, and self-financial support.

### Strengths of the study

To the best of our knowledge, this was the first study on the gambling and gambling disorder in this part of the country. It also has large sample size, which gave it advantage over other studies with smaller sample sizes.

### Limitations of the study

The data were obtained from self-reported questionnaire, which is not free of response bias. Also, this study was a cross-sectional study and thus cannot establish causality and direction of relationship. The study result may also not be generalisable to the larger Nigerian population as it was conducted among tertiary institution students.
